# Neurocutaneous Melanosis with Meningeal Melanocytosis: A Rare Case of Intracranial Hypertension and Cutaneous Manifestations

**DOI:** 10.3390/life14010139

**Published:** 2024-01-18

**Authors:** Hsien-Chung Chen, Tsung-I Hsu, Tsu-Yi Chao, Shun-Tai Yang

**Affiliations:** 1 Ph.D. Program in Medical Neuroscience, College of Medical Science and Technology, Taipei Medical University and National Health Research Institutes, Taipei 110, Taiwan; d620111001@tmu.edu.tw (H.-C.C.); dabiemhsu@tmu.edu.tw (T.-I.H.); 2 Department of Neurosurgery, Shuang Ho Hospital, Taipei Medical University, Taipei 110, Taiwan; 3 Taipei Neuroscience Institute, Taipei Medical University, Taipei 110, Taiwan; 4 TMU Research Center of Neuroscience, Taipei Medical University, Taipei 110, Taiwan; 5 International Master Program in Medical Neuroscience, College of Medical Science and Technology, Taipei Medical University, Taipei 110, Taiwan; 6 TMU Research Center of Cancer Translational Medicine, Taipei 110, Taiwan; 7 Ph.D. Program in Drug Discovery and Development Industry, College of Pharmacy, Taipei Medical University, Taipei 110, Taiwan; 8 Cancer Center, Attending Physician, Division of Hematology-Oncology, Shuang Ho Hospital, Taipei Medical University, Taipei 110, Taiwan; 10575@s.tmu.edu.tw; 9 Graduate Institute of Medical Sciences, College of Medicine, Taipei Medical University, Taipei 110, Taiwan

**Keywords:** melanocytic nevus cell nests, neurocutaneous melanosis, ventriculoperitoneal shunt

## Abstract

A 50-year-old male presented to the emergency room after experiencing sudden right upper limb facial numbness and dysphasia, followed by full recovery. A brain CT scan showed hyperdense lesions within the left hemispheric sulcus, which raised suspicion of spontaneous subarachnoid hemorrhage. A T1-weighted MRI showed multiple tiny leptomeningeal enhancements in the same area, and a digital subtraction angiography showed no signs of vascular abnormality. Cerebrospinal fluid cytology revealed atypical melanin-containing cells with minimal pleomorphism. One month later, the patient developed sixth nerve palsy, which was determined to be due to intracranial hypertension. Multiple giant nevi on the legs, trunk, and scalp were also observed. A skin biopsy showed well-defined and symmetrical proliferation of melanocytic nevus cell nests in the dermis. An open biopsy was performed due to the suspicious leptomeningeal lesions, which surprisingly revealed diffuse and thick black-colored tissue infiltration of the leptomeninges. Pathology confirmed the diagnosis of meningeal melanocytosis. A ventriculoperitoneal shunt was then placed, and the patient’s neurological symptoms gradually improved. Based on the presence of multiple giant nevi on the patient’s skin and the finding of diffuse meningeal melanocytosis during the open biopsy, the patient was diagnosed with neurocutaneous melanosis. The patient received 6 cycles triweekly of Ipilimumab and Nivolumab 8 months after initial diagnosis. Unfortunately, the disease progressed and the patient passed away 14 months after initial diagnosis.

## 1. Background

Neurocutaneous melanosis (NCM) is an exceptionally rare congenital neurocutaneous syndrome primarily affecting children, with adult cases being exceedingly uncommon. This syndrome is characterized by a complex combination of skin and central nervous system abnormalities, most notably the presence of congenital melanocytic nevi along with the development of benign or malignant melanoma in the central nervous system. NCM is believed to stem from embryological defects in the migration of melanoblasts from the neural crest to the leptomeninges and skin. The first documented case of NCM dates back to 1861 when it was described by Rokitansky. Typically, patients with NCM exhibit neurological symptoms at an early age, often leading to a grim prognosis regardless of whether the central nervous system tumors are benign or malignant. Sadly, most cases result in a fatality within three years of symptom onset [1,2,3,4].

NCM arises from errors during embryonic neuroectoderm development, particularly affecting the neural crest. It manifests as giant congenital nevi on the skin and melanocyte hyperplasia within the central nervous system. Unfortunately, there is currently no effective treatment for NCM, and the prognosis is extremely poor, especially due to the propensity of meningeal lesions to undergo malignant transformations. In children, NCM typically leads to neurological symptoms attributed to either intracranial malignant melanoma or the continued growth of benign melanin cells, often resulting in death before the third year of life. Consequently, reports of adults with NCM are exceptionally rare. Given its rarity and grave prognosis, NCM remains a challenging condition for both patients and healthcare providers [4].

## 2. Case Presentation

**Figure 1:**
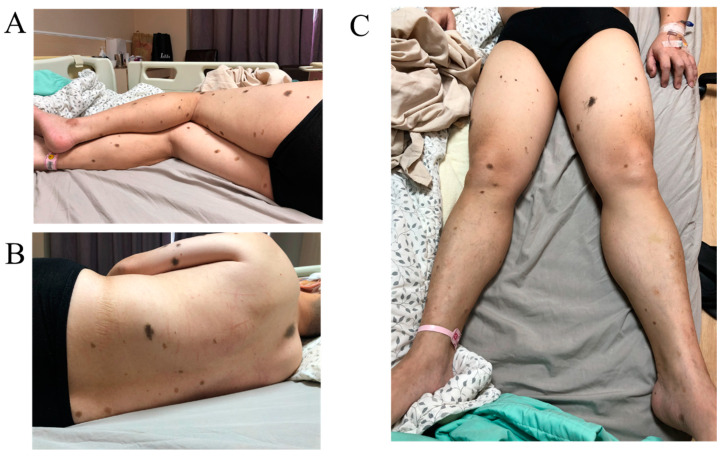
Physical examination found multiple nevus all over the body. (**A**) Nevus in the lower limbs. (**B**) Nevus in the back. (**C**) Nevus in the front of lower limbs.

The 50-year-old male patient presents with a medical history of being an HBV carrier and no known history of smoking, hypertension, or heart disease. The medical history of the patient includes being an HBV carrier, which has no established correlation with melanosis. He experienced a transient episode of neurological symptoms characterized by sudden-onset right arm and right face numbness, along with limited range of motion in the left eye, which lasted for approximately 10 min and subsequently resolved completely. Notably, there is no reported muscle weakness or history of trauma. Additionally, the patient exhibits the presence of multiple nevi with hair on various regions of the body (Figure 1), including all four limbs, the upper back, and the scalp. Given the constellation of symptoms and the cutaneous findings, further neurological evaluation is warranted to assess the potential underlying etiology, including the consideration of conditions such as NCM or other neurological disorders. The patient presented with additional concerning symptoms one month after the initial evaluation, including progressive left eye lateral gaze limitation, escalating headache, and dizziness. Importantly, there is no reported development of hand clumsiness at this time. These new neurological symptoms, especially the limitation of left eye movement and worsening headache with dizziness, may indicate a potential progression or change in the underlying condition. Further neurological assessment and imaging studies may be essential to identify and manage the cause of these evolving symptoms effectively. We did not initially consider NCM. It was only during the second hospitalization that a thorough examination was conducted. Dr. Yang had prior experience diagnosing this condition, which led to the suspicion of this disease. Ultimately, surgery confirmed the diagnosis.

**Figure 2:**
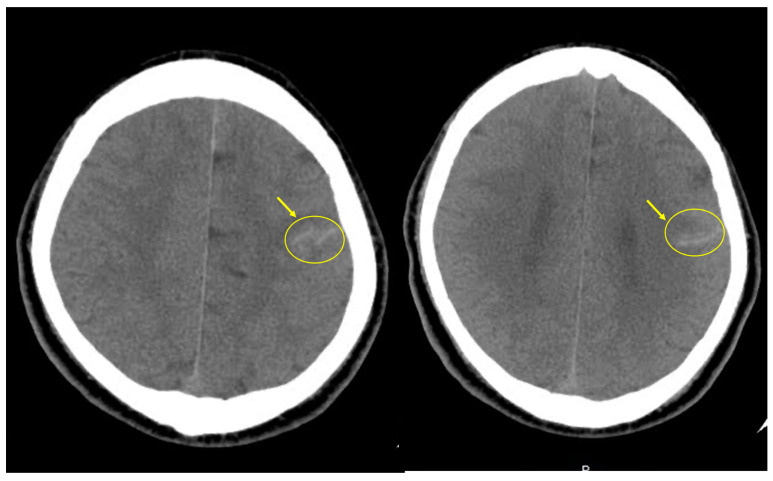
Suspicious of subarachnoid hemorrhage in initial brain CT. Lesions were marked with a yellow circle.

**Figure 3:**
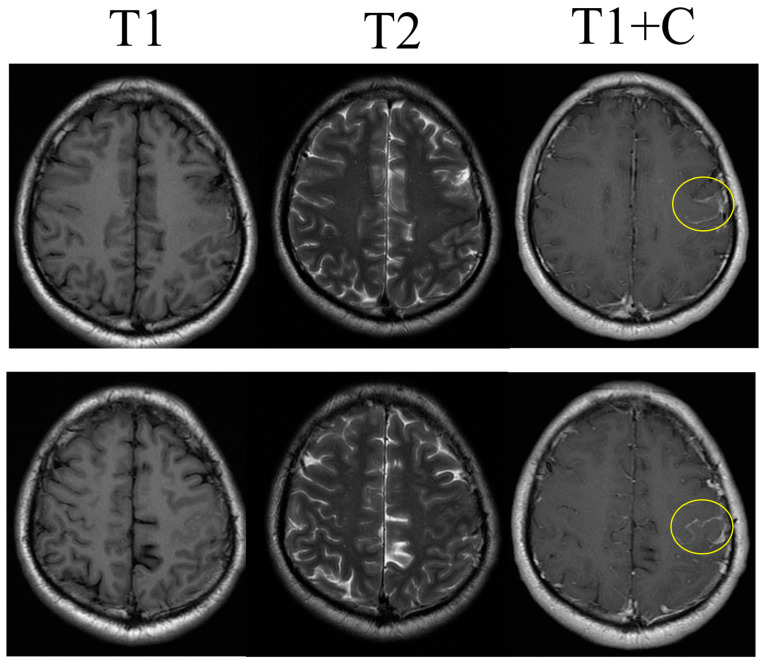
Leptomenigeal enhancement in initial brain MRI. T1 and T2 images. Lesions marked with the yellow circle were identified in T1 image with contrast agent administration.

**Figure 4:**
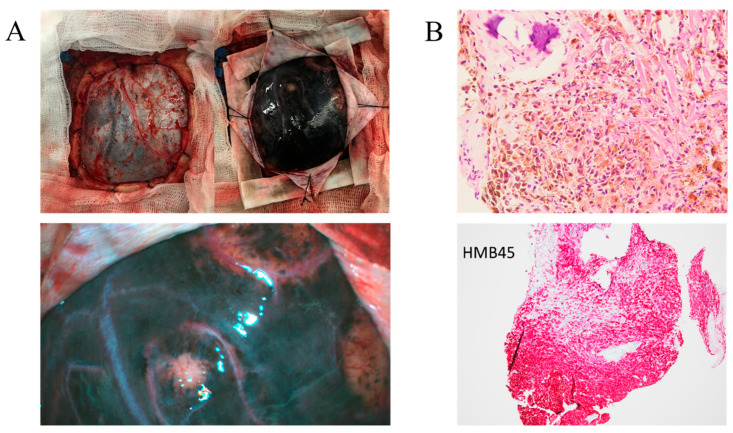
The open biopsy procedure was notable for the tumor’s propensity to bleed easily upon touch (**A**), which is consistent with its highly vascularized nature. (**B**) Upper: Melan A staining; Lower: HMB45 staining.

The CT and MRI findings reveal abnormal enhancement in the left hemispheric region, raising several diagnostic possibilities, including spontaneous subarachnoid hemorrhage (Figure 2), leptomeningeal metastasis, or leptomeningeal carcinomatosis (Figure 3). The operative biopsy results reveal a concerning picture of diffuse and thick black-colored tissue infiltration in the leptomeninges, with notable involvement of cortical vessels. These infiltrative characteristics, along with the propensity for easy bleeding within the tumor, underscore the aggressive and highly vascularized nature of the lesion (Figure 4A). Upon histological examination, the specimens appeared grossly as soft, black lesions. Microscopically, the samples revealed numerous pigmented cells characterized by uniform ovoid nuclei. Notably, bleached H&E sections exhibited no evidence of necrosis, mitosis, or cellular pleomorphism. Immunohistochemical analysis revealed positive staining for Melan A and HMB45, indicative of melanocytic differentiation (Figure 4B). An attempt to perform an immunohistochemical stain for BRAF V600E was challenging due to the heavy pigmentation of the tissue, making interpretation difficult. In summary, the histological findings are consistent with the possibility of meningeal melanocytosis, a diagnosis that warrants further clinical correlation and evaluation.

Cerebrospinal fluid (CSF) cytology results reveal the presence of atypical melanin-containing cells with minimal nuclear pleomorphism but notable nucleoli, both as individual cells and in clusters. Immunohistochemical staining for melan A has shown positive results, confirming the presence of atypical melanocytic cells in the CSF. In addition, positive expression for HMB45, indicative of melanocytic differentiation, was detected in the specimen. Taken together, these findings strongly suggest the presence of atypical melanocytic cells in the CSF, indicating a potential central nervous system involvement of melanocytic origin. Additionally, immune-related parameters were assessed, encompassing white blood cell count, neutrophil, lymphocyte, and eosinophil levels, along with Pandy’s test for CSF. These evaluations ruled out symptoms related to infection. Further diagnostic evaluations and discussions with relevant specialists are warranted to determine the extent of the condition and plan an appropriate course of action.

Due to limited specimen availability, a liquid biopsy was conducted using FoudationOne^®®^Liquid CDx, revealing specific genomic findings, including *DNMT3A* mutations (R771Q and R635W) and an *SDHA* mutation (M1V). However, no clinically relevant single-nucleotide variant or copy number variation (CNV) associated with the described phenotype or clinical suspicion was detected using whole exome sequencing by CentoXome^®®^. Furthermore, no clinically significant variants related to the described phenotype were identified within the mitochondrial genome through sequencing.

**Figure 5:**
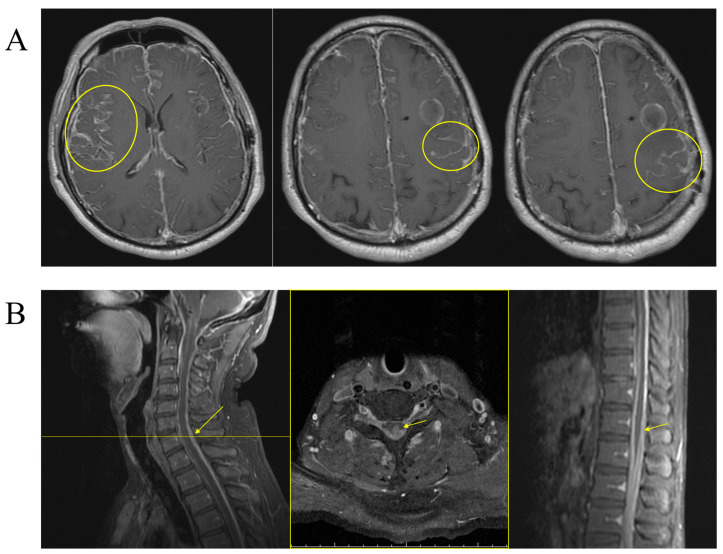
Disease progression in 8-month follow-up MRI. (**A**) Brain MRI images display lesions encircled in yellow. (**B**) Spinal MRI images presented as follows: Left image showcases a cervical spine MRI with a lesion denoted by a yellow arrow. The middle image is a transverse cut of the cervical region, corresponding to the position of the yellow horizontal line in the left image, where the lesion is again highlighted with a yellow arrow. The right image is a thoracic spine MRI with a lesion indicated by a yellow arrow.

We express profound concern as we observe the patient’s recent onset of cauda equina syndrome, occurring 8 months after the initial diagnosis and attributed to spinal NCM (Figure 5). The patient’s medical history includes extensive leptomeningeal and spinal cord involvement, along with a prior left frontal melanoma for which a craniotomy with minimal tumor removal and ventriculoperitoneal (VP) shunt placement were performed in May 2021. Despite receiving 6 cycles of triweekly Ipilimumab and Nivolumab treatment 8 months after the initial diagnosis, it is disheartening to learn that the disease continued to progress. The condition of the patient ultimately led to their passing 14 months after the initial diagnosis. This serves as a poignant reminder of the complexities and challenges posed by aggressive diseases like NCM and the need for ongoing research and improved treatment options in the field of oncology.

## 3. Discussion and Conclusions

NCM is a rare and challenging condition characterized by the presence of congenital melanocytic nevi and the potential for central nervous system (CNS) involvement. A critical aspect of NCM management is distinguishing between benign melanosis and malignant transformation, particularly leptomeningeal melanoma [5]. Imaging techniques, such as MRI, may be limited in their ability to definitively differentiate between benign and malignant entities, necessitating careful clinical evaluation. Positron emission tomography/computed tomography (PET/CT) with radiotracers like [F-18]2-fluoro-2-deoxyglucose (FDG) and C-11-methionine has been suggested for diagnosing malignant transformation [6,7], although it may not always provide conclusive results. In some cases, neurological symptoms, imaging findings such as adjacent edema, or rapid melanosis growth may be more indicative of malignancy. Importantly, symptomatic NCM is associated with a poor prognosis regardless of histological malignancy presence or absence [8,9,10].

The risk of malignant transformation in NCM is particularly associated with large congenital melanocytic nevi, especially those located on the torso, often referred to as “bathing trunk” distribution [11]. These nevi carry a risk of 2.5% to 5%, with the highest risk in the first decade of life [12]. Furthermore, large congenital melanocytic nevi, particularly those accompanied by multiple satellite nevi, are closely associated with the development of NCM, which poses significant risks of primary CNS melanoma and death [13]. Interestingly, our study suggests that the location of giant congenital melanotic nevi on the scalp or face may have an association with the absence of evident MRI signs of CNS melanosis, although the reasons behind this association remain unclear. The risk of patients with initially negative imaging evidence of melanosis later developing melanosis or malignant melanoma remains unknown, underscoring the need for long-term monitoring and further research in this area [11,13].

Pathological analysis plays a crucial role in confirming melanoma diagnosis, with markers such as S-100, MelanA, and HMB-45 being vital for differentiation. The cell proliferating nuclear antigen-positive rate is also significant for distinguishing malignant melanoma from benign melanoma. In one case, pathological examination revealed abundant melanin granules within tumor cells, characterized by multiple horns or fusiform shapes. Large oval nuclei, nuclear fission elements, nucleated giant cells, and a high mitotic index were observed. Immunohistochemistry confirmed positivity for HMB-45, MelanA, and S-100, further substantiating the melanoma diagnosis [14,15,16]. While it is true that the giant nevi on the limbs and trunk were not biopsied, the diagnosis was supported by the presence of neurological symptoms in addition to the cutaneous manifestations. These symptoms were suggestive of a possible neurocutaneous disorder. Upon surgical intervention, we were able to confirm melanosis within the central nervous system (Figure 4A). Further, the biopsy and subsequent histological analysis of the brain tissue revealed the presence of melanocytes, corroborating the diagnosis of NCM.

Patients with NCM can be categorized into symptomatic and asymptomatic groups. Symptomatic cases often manifest in two distinct peaks, with the first occurring around the age of 3, marked by increased intracranial pressure, seizures, hydrocephalus, cranial nerve dysfunction, hemiplegia, and developmental delays [11,17]. The second peak emerges around age 20, typically involving symptoms such as headaches, seizures, or mental disturbances [10,17]. Mortality rates and survival data in NCM are challenging to ascertain due to the rarity of the condition and the need for larger sample sizes in follow-up studies. In adult NCM cases, a notable gender disparity exists, with approximately twice as many males as females. While this difference may not be statistically significant given the small patient pool, further research is required to understand potential correlations between gender, estrogen levels, and the incidence of adult NCM [17,18].

Diagnosing and treating NCM is particularly complex. The condition is more common in pediatric populations, which makes adult cases exceptionally rare and thus less studied. One of the primary diagnostic challenges is the identification of NCM in adults due to the atypical presentation of symptoms and the low index of suspicion among clinicians. Neurological symptoms, such as intracranial hypertension, seizures, and hydrocephalus, are not specific to NCM and can be indicative of a variety of other central nervous system pathologies, which can lead to misdiagnosis or delayed diagnosis [10,11,18,19]. Imaging techniques, although essential for diagnosis, have their limitations. MRI, the most informative non-invasive diagnostic tool for NCM, can reveal intracranial and intraspinal melanocytic lesions, but distinguishing between diffuse melanocytosis, meningeal melanocytoma, and malignant melanoma solely based on imaging characteristics is challenging [18,19]. Histological examination is necessary to confirm the diagnosis, which can be invasive and is not without risk. Even when imaging and histopathology point to NCM, the presence of melanin in the lesions can vary, altering the appearance on MRI and complicating the interpretation. Additionally, the imaging might not detect very small lesions when neurological symptoms are absent, further delaying diagnosis. 

Regarding treatment, the options are limited and generally have poor outcomes. Surgical resection is recommended for localized lesions, but the intricate and vascular nature of melanomas can render complete removal impossible, particularly when the melanoma is diffuse. Adjuvant therapies, such as chemotherapy and radiotherapy, have been used, but their effectiveness is questionable, and they are often not curative [10,19]. The highly vascular melanomas also pose a surgical risk, and the potential for diffuse lesions complicates the possibility of achieving clear margins during resection [11]. The prognosis for NCM is typically poor, as even benign melanocytosis can lead to severe complications and an increased risk of malignant transformation [5,18,19]. The risk is higher when lesions are located in areas like the head, neck, back, and buttocks and are accompanied by satellite nevi. The previous study suggests that while the congenital giant nevi associated with NCM can be observed at birth, the neurological symptoms that lead to the diagnosis often do not appear until later, sometimes not until adulthood, which further complicates the clinical picture [19]. Additionally, treatments like ventriculoperitoneal shunt surgery for hydrocephalus associated with NCM can be compromised by melanin cells blocking the shunt, and there is a risk of peritoneal metastasis [18]. Follow-up treatment for NCM is also complicated by the sensitivity of the subarachnoid melanin cells to therapeutic interventions, and the quality of life for these patients is a significant concern.

In conclusion, NCM remains a complex and enigmatic condition that poses diagnostic and therapeutic challenges. Accurate diagnosis, vigilant monitoring, and further research into more aggressive treatment modalities are essential to improve outcomes for patients with this rare disorder.

## Data Availability

Data are contained within the article.

## Abbreviations

NCM	Neurocutaneous melanosis
CSF	Cerebrospinal fluid
VP	Ventriculoperitoneal
CNS	Central nervous system
PET/CT	Positron emission tomography/computed tomography
FDG	[F-18]2-fluoro-2-deoxyglucose
